# Discovery of Novel Genetic Risk Loci for Acute Central Serous Chorioretinopathy and Genetic Pleiotropic Effect With Age-Related Macular Degeneration

**DOI:** 10.3389/fcell.2021.696885

**Published:** 2021-08-20

**Authors:** Lei Feng, Si Chen, Huatuo Dai, Rajkumar Dorajoo, Jianjun Liu, Jinfeng Kong, Xianyong Yin, Yunqing Ren

**Affiliations:** ^1^Eye Center, The Second Affiliated Hospital of Zhejiang University School of Medicine, Hangzhou, China; ^2^Department of Ophthalmology, Jinshan Branch of Shanghai Sixth People’s Hospital, Shanghai, China; ^3^Department of Dermatology, The Children’s Hospital, Zhejiang University School of Medicine, National Clinical Research Center for Child Health, Hangzhou, China; ^4^Department of Dermatology, The Second Affiliated Hospital of Zhejiang University School of Medicine, Hangzhou, China; ^5^Genome Institute of Singapore, Agency for Science, Technology and Research, Singapore, Singapore; ^6^Department of Medicine, Yong Loo Lin School of Medicine, National University of Singapore, Singapore, Singapore; ^7^Department of Biostatistics and Center for Statistical Genetics, University of Michigan School of Public Health, Ann Arbor, MI, United States

**Keywords:** central serous chorioretinopathy, gene, association, pleiotropic effect, risk loci, age-related macular degeneration

## Abstract

**Background:**

Central serous chorioretinopathy (CSC) is a severe and heterogeneous chorioretinal disorder. Shared clinical manifestations between CSC and age-related macular degeneration (AMD) and the confirmation of CFH as genetic risk locus for both CSC and AMD suggest possible common pathophysiologic mechanisms between two diseases.

**Methods:**

To advance the understanding of genetic susceptibility of CSC and further investigate genetic pleiotropy between CSC and AMD, we performed genetic association analysis of 38 AMD-associated single nucleotide polymorphisms (SNPs) in a Chinese CSC cohort, consisting of 464 patients and 548 matched healthy controls.

**Results:**

Twelve SNPs were found to be associated with CSC at nominal significance (*p* < 0.05), and four SNPs on chromosomes 1, 4, and 15 showed strong associations whose evidences surpassed Bonferroni (BF)-corrected significance [rs1410996, odds ratios (OR) = 1.47, *p* = 2.37 × 10^–5^; rs1329428, *OR* = 1.40, *p* = 3.32 × 10^–4^; rs4698775, *OR* = 1.45, *p* = 2.20 × 10^–4^; and rs2043085, *OR* = 1.44, *p* = 1.91 × 10^–4^]. While the genetic risk effects of rs1410996 and rs1329428 (within the well-established locus CFH) are correlated (due to high LD), rs4698775 on chromosome 4 and rs2043085 on chromosome 15 are novel risk loci for CSC. Polygenetic risk score (PRS) constructed by using three independent SNPs (rs1410996, rs4698775, and rs2043085) showed highly significant association with CSC (*p* = 2.10 × 10^–7^), with the top 10% of subjects with high PRS showing 6.39 times higher risk than the bottom 10% of subjects with lowest PRS. Three SNPs were also found to be associated with clinic manifestations of CSC patients. In addition, by comparing the genetic effects (ORs) of these 38 SNPs between CSC and AMD, our study revealed significant, but complex genetic pleiotropic effect between the two diseases.

**Conclusion:**

By discovering two novel genetic risk loci and revealing significant genetic pleiotropic effect between CSC and AMD, the current study has provided novel insights into the role of genetic composition in the pathogenesis of CSC.

## Introduction

Central serous chorioretinopathy (CSC) is a common chorioretinal disorder characterized by serous sensorial retinal detachment (Balaratnasingam et al., 2016). It is now recognized as the fourth most incident eye disorder after age-related macular degeneration (AMD), diabetic retinopathy, and branch retinal vein occlusion among non-surgical retinopathies (Wang et al., 2008). CSC occurs most frequently in midlife, and the prevalence in men is nearly six times higher than in women (Wang et al., 2008; Liew et al., 2013). The etiology of CSC remains unclear, but several factors have been implicated in the disease development including administration of corticosteroids, hypercortisolism, stress, altered plasma cytokine levels, and genetic risk factors (Daruich et al., 2015; Karska-Basta et al., 2020, 2021a, 2021b). CSC patients cluster in families (Oosterhuis, 1996; Weenink et al., 2001), and the prevalence of CSC varies in different ethnic populations, with higher prevalence in Asians than in Caucasians and African Americans (Desai et al., 2003; How and Koh, 2006; Kitzmann et al., 2008; Tsai et al., 2013), suggesting that genetic compositions may contribute to the risk of CSC.

Two main subtypes of CSC, acute and chronic CSC, show distinct clinical presentations in terms of the episode duration, extent of retinal abnormalities, and final vision recovery (Daruich et al., 2015). In comparison with chronic CSC, patients with acute CSC characteristically show a sudden onset of obvious visual deterioration and relatively short course within 6 months (Daruich et al., 2017). In multivariate regression analyses, age of onset, duration of disease, and hyperopia were positively associated with the risk of chronic disease rather than acute CSC (Ersoz et al., 2018). Although most of acute CSC patients have been described to be self-limiting, some patients still suffer recurrences or progress to a chronic condition, indicating interindividual variation of risk and clinical course in acute CSC. These clinical differences suggest that some aspects of underlying molecular pathogenesis might be different between acute and chronic CSCs.

Genetic association studies have identified several genetic variants that are significantly associated with CSC, including single nucleotide polymorphisms (SNPs) in the complement factor H (CFH), solute carrier family 7 member 5 (SLC7A5), age-related maculopathy susceptibility 2 (ARMS2), nuclear receptor subfamily 3 group C member 2 (NR3C2), cadherin 5 (CDH5), and TNF receptor superfamily member 10a (TNFRSF10A) gene, as well as copy number variations in the complement C4B gene (Miki et al., 2014, 2018; Schubert et al., 2014; Breukink et al., 2015; de Jong et al., 2015; van Dijk et al., 2017; Schellevis et al., 2018; Hosoda et al., 2019). However, previous studies focused primarily on chronic CSC, and mainly in western and Japanese populations (van Dijk et al., 2017; Miki et al., 2018; Schellevis et al., 2018). The genetic association study in acute CSC may help to identify genetic factors responsible for clinical variability between acute and chronic CSC, and among acute CSC patients. In addition, the diverse prevalence of CSC across different regions and countries also suggests a possible variation of genetic susceptibility across different ethnic backgrounds (Desai et al., 2003; Tsai et al., 2013). As a result, study in Chinese populations would have the potential to identify novel genetic risk loci and elucidate the genetic heterogeneity of CSC across different populations.

Notably, CFH gene, as a well-established AMD susceptibility gene, is the only genetic association locus for CSC that has been replicated in multiple independent studies so far (Miki et al., 2014; de Jong et al., 2015; Schellevis et al., 2018; Karkhaneh et al., 2021). Interestingly, several manifestations of CSC, such as retinal pigment epithelium (RPE) disruption and neurosensory retinal detachment in macula, are also frequently observed in AMD patients. Those eyes with CSC were documented to be at higher risk of developing AMD even after spontaneous resolution of CSC (Hosoda et al., 2018). Together, these suggest that CSC and AMD may share some common genetic and pathophysiologic background. To investigate AMD-associated genetic risk variants in CSC patients by a genetic association analysis is a good strategy to elucidate genetic susceptibility for CSC and to explore genetic pleiotropic effect between CSC and AMD.

Here, we performed a genetic study to evaluate the association of 38 known AMD-associated SNPs with the risk of acute CSC in a Chinese population. We further investigated the impact of significantly associated risk variants on the clinical manifestations in CSC patients and compared the genetic effects of these loci between CSC and AMD. These findings shed light on the genetic susceptibility of CSC in Chinese populations as well as genetic pleiotropic effect between CSC and AMD.

## Materials and Methods

### Study Participants

A total of 464 Chinese patients were recruited, all of whom had their first episode of idiopathic acute CSC. All patients were enrolled from the Eye Center, the Second Affiliated Hospital, School of Medicine, Zhejiang University, China, between January 2014 and April 2017. Each patient underwent an extensive ophthalmologic examination including fundoscopy, spectral-domain optical coherence tomography (SD-OCT, Zeiss Cirrus OCT 5000, Germany), fundus fluorescein angiography (FFA, Spectralis HRA-II, Germany), and indocyanine green angiography (ICGA, Spectralis HRA-II, Germany). Inclusion criteria in our study were initial presentation of less than 20 days after eye symptoms onset, the presence of subretinal fluid (SRF) in OCT, focal leakage spot on FFA and corresponding hyperfluorescence on ICGA, and without any atrophic RPE changes. Consensus diagnosis of acute CSC was performed by two senior ophthalmologists (LF and SC). We excluded chronic CSC patients that were diagnosed with chronic SRF leakage, with extensive multifocal atrophic RPE changes and/or disease duration of more than 3 months. In addition, we excluded patients with any evidence of AMD, polypoidal choroidal vasculopathy, retinal vascular diseases, diabetic retinopathy, spherical error higher than 3D, intraocular surgery, and laser history. A total of 548 healthy controls were included in the study. Any participants were excluded from the controls if they were (1) with any clinical manifestations of visual impairment, visual distortion, and dyslexia; (2) with any early AMD symptoms in fundus examination, such as pigmentation disorder and atrophy in macular area; (3) with CSC manifestations detected by OCT and FFA; (4) with other ocular and systemic diseases. All the study subjects were of Chinese origin. Each participant provided written informed consent. This study was approved by the Ethics Committee of Zhejiang University School of Medicine and was conducted according to the Declaration of Helsinki.

### DNA Isolation and Genotyping

Ethylenediamine tetracetic acid (EDTA)-anticoagulated venous blood samples were collected from each participant, and then genomic DNA from peripheral blood leukocytes were extracted by standard procedures using AxyPrep-96 Blood Genomic DNA Kits (Axygen, Union City, CA, United States). We selected 47 SNPs that have been previously reported for the risk of AMD through manual literature review (Supplementary Table 1) and genotyped them in 464 acute CSC patients and 548 controls by SNaPshot Multiplex Kit (Applied Biosystems Co., San Francisco, CA, United States). All procedures were performed according to the manufacturer’s instructions.

### Quality Control and Statistical Analysis

Three SNPs with minor allele frequency (MAF) <1%, or Hardy-Weinberg disequilibrium in the controls (*p* < 10^–6^, *n* = 6 SNPs) were excluded from further analysis. In addition, six samples were excluded due to low genotyping call rates (i.e., call rate < 90%). We tested the single-variant association with the risk of CSC using logistic regression with gender and age as covariates in Plink v1.90 (Chang et al., 2015). *p* < 0.001 (0.05/38) was adopted as the significance threshold after Bonferroni correction for multiple testing. We also evaluated the association between the significant risk variants and clinical manifestations relevant to FFA (FFA-traits) and OCT diagnoses (OCT-traits) in the CSC patients through logistic regression (FFA) or linear regression (OCT) in Plink v1.90. We included three covariates, including gender, eye, and age at recruitment, in FFA-traits association analysis, while adjusting an additional covariate of OCT equipment in OCT-traits association tests. To evaluate the cumulative effect of genetic variants on CSC risk, we computed a polygenic risk score (PRS) for each participant through adding up their genotypes for the three independently significant SNPs which were weighted by their corresponding association effect sizes. We then grouped the subjects into four quartiles by their PRSs in healthy controls. The cumulative risk effects were assessed by estimating the odd ratios against the first quartile through logistic regression test.

For the gene ontology (GO) term enrichment analysis, known genes within the 10-kb regions of 12 SNPs shared between CSC and AMD were mapped using the SNP2GENE function in Functional Mapping and Annotation of Genome Wide Association Studies (FUMA) (Watanabe et al., 2017). Gene-set enrichments in GO terms were subsequently performed for the genes using the GENE2FUNC function in FUMA. In gene-set enrichments, tests for overrepresentation of the genes were performed using hypergeometric tests. All gene-set enrichment *p*-values were adjusted using Benjamini-Hochberg (B-H) correction for multiple tests.

## Results

### Characteristics of Study Subjects

**FIGURE 1:**
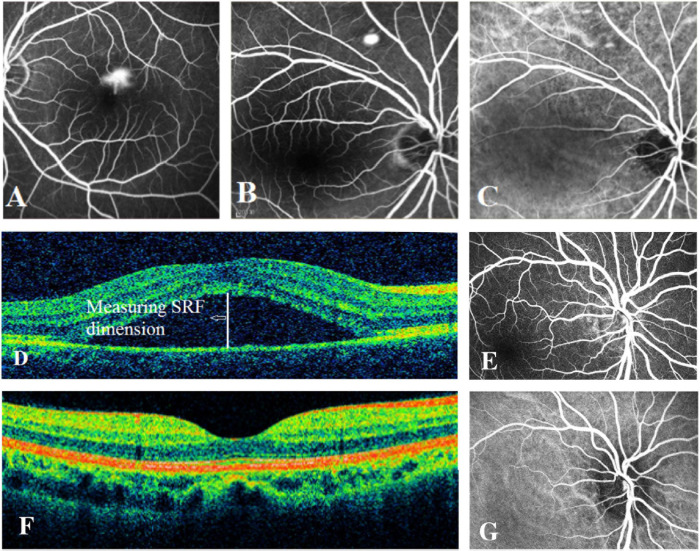
Examples of fundus fluorescein angiography (FFA), indocyanine green (ICG) angiography, and spectral-domain optical coherence tomography (SD-OCT) in individuals with acute CSC and controls. **(A)** The left eye of an individual with a typical smokestack leakage point on FFA. **(B)** The right eye of an individual with a typical inkblot-like leakage point on FFA. **(C)** The ICGA image of the same individual as in panel **(B)**, showing choroidal vascular dilatation. **(D)** An obvious central subretinal fluid (SRF) between retinal neurepithelium layer and retinal pigment epithelium layer on OCT. **(E)** The FFA image of one of the controls showing no leakage point. **(F)** The OCT image of one of the controls showing no subretinal fluid. **(G)** The normal ICGA image of one of the controls.

In our study, 464 acute CSC patients and 548 controls were recruited. The cases (average age = 44.45 ± 7.04 years) and controls (average age = 46.80 ± 14.53 years) were matched on age, but there is a moderate difference in gender ratio between the cases (81.03% being males) and controls (62.22% being males). The clinical features of acute CSC patients are shown in Figure 1. The averages of horizontal and vertical dimensions of SRF were 280.06 ± 139.52 μm and 290.90 ± 146.98 μm, respectively. The averages of central subfield thickness and volume were 451.27 ± 146.46 μm and 11.56 ± 1.78 μm, respectively. FFA imaging characteristics varied among patients (Figure 1): 1 leakage point in 319 eyes (69.96%), 2 leakage points in 137 eyes (30.04%), inkblot-like leakage in 360 eyes (78.95%), and smokestack leakage in 96 eyes (21.05%).

### Association Analysis of AMD Loci in Acute CSC

**FIGURE 2:**
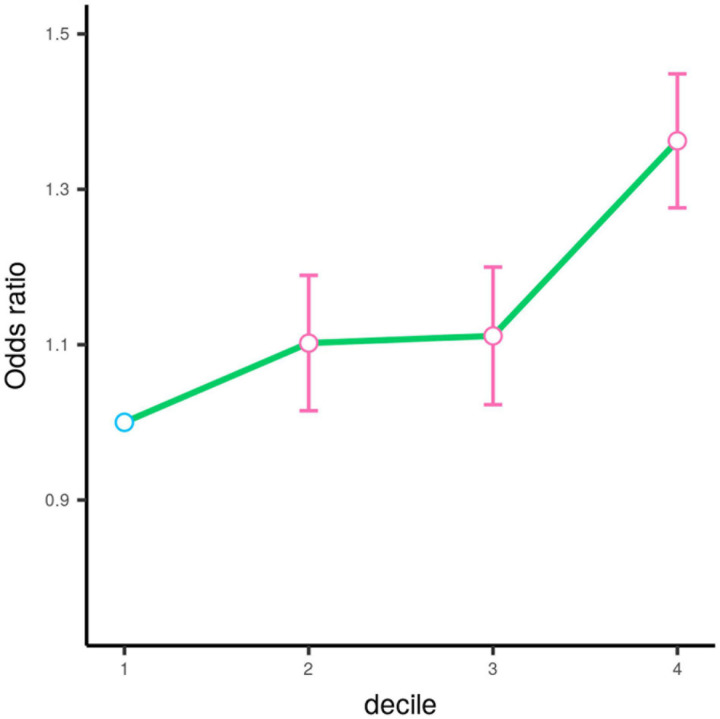
Odds ratios (OR) by the quartile of polygenic risk score estimated based on the top three SNPs. The circle denotes the estimated value of odds ratio. The error bar represents the 95% confidence interval for the odds ratio estimation. The circle is colored in pink (*p* < 0.05) and blue (*p* > 0.05) according to its corresponding respective *p*-value.

We tested single-variant association between 38 known AMD-risk SNPs and the risk of CSC in 459 cases and 547 controls. Of the 38 SNPs tested, 12 SNPs showed association at nominal significance (*p* < 0.05; Table 1 and Supplementary Table 1), and 4 SNPs on chromosomes 1, 4, and 15 showed strong association with Bonferroni (BF)-corrected significance (Chr 1: rs1410996, *OR* = 1.47, *p* = 2.37 × 10^–5^; Chr 1: rs1329428, *OR* = 1.40, *p* = 3.32 × 10^–4^; Chr 4: rs4698775, *OR* = 1.45, *p* = 2.20 × 10^–4^; and Chr 15: rs2043085, *OR* = 1.44, *p* = 1.91 × 10^–4^). Rs1410996 and rs1329428 on chromosome 1 are in strong linkage disequilibrium (*r*^2^ = 0.94); the association at SNP rs1329428 diminished after conditioning on rs1410996 (*p* > 0.05). Pairwise interaction analyses among the three SNPs were also conducted using logistic regression. We only detected interaction between rs1410996 and rs4698775 (*p* = 0.038), which is, however, not statistically significant after correction for multiple pair-wise interaction testing, suggesting that the genetic association effects at these SNPs are largely additive. Our results also suggested that the genetic association effects at rs1410996, rs4698775, and rs2043085 followed a recessive mode of inheritance (Table 2). Next, these three SNPs were used to calculate a PRS for each subject. The PRS showed a highly significant association with CSC (*p* = 2.10 × 10^–7^), with the top 10% of subjects with highest PRS showing 6.39 times higher risk than the bottom 10% of subjects with lowest PRS. In comparison with the first quartile, the second, third, and fourth quartiles of PRS conferred significant cumulative risk effect (*OR* = 1.10, *p* = 0.03; *OR* = 1.11, *p* = 0.02; *OR* = 1.33, *p* = 4.18 × 10^–11^; Figure 2).

**TABLE 1:** Association results for 12 AMD-known SNPs with nominal significance in acute CSC in Han Chinese population.

**SNP**	**CHR**	**BP**	**Gene**	**A1/A2^a^**	**EAF**		**SE**	***P*-value**	**OR-CSC**	**OR-AMD**
					**Case**	**Control**				
rs1410996	1	196,696,933	*CFH*	A/G	0.52	0.41	0.09	2.37E−05	1.47	0.37 (Yu et al., 2011)
rs1329428	1	196,702,810	*CFH*	T/C	0.52	0.43	0.09	3.32E−04	1.40	0.36 (Kopplin et al., 2010)
rs2043085	15	58,680,954	*LIPC*	A/G	0.55	0.46	0.10	1.91E−04	1.44	1.15 (Fritsche et al., 2016)
rs4698775	4	110,590,479	*MCUB-PLA2G12A-CFI*	G/T	0.29	0.21	0.10	2.20E−04	1.45	1.14 (Fritsche et al., 2013)
rs429358	19	45,411,941	*APOE*	C/T	0.03	0.06	0.24	8.39E−03	0.53	0.70 (Fritsche et al., 2016)
rs10507047	12	95,604,290	*FGD6*	C/T	0.34	0.29	0.10	1.85E−02	1.26	0.87 (Cheng et al., 2015)
rs1142	7	104,756,326	*KMT2E*, *SRPK2*	T/C	0.33	0.29	0.10	2.30E−02	1.26	1.11 (Fritsche et al., 2016)
rs4420638	19	45,422,946	*APOE*	G/A	0.08	0.11	0.16	2.56E−02	0.70	0.77 (Fritsche et al., 2013)
rs1713985	4	57,786,450	*NOA1*, *REST*, *IGFBP7*, *POLR2B*	G/T	0.23	0.27	0.11	2.86E−02	0.79	1.30 (Arakawa et al., 2011)
rs10781182	9	76,617,720	*RORB*	G/T	0.29	0.24	0.11	4.11E−02	1.26	0.90 (Fritsche et al., 2016)
rs3812111	6	116,443,735	*COL10A1*	A/T	0.25	0.19	0.11	4.12E−02	1.24	0.91 (Fritsche et al., 2013)
rs2740488	9	107,661,742	*ABCA1*	C/A	0.26	0.22	0.12	4.98E−02	1.26	0.90 (Fritsche et al., 2016)

*SNP, single nucleotide polymorphism; CHR, chromosome; BP, base pair in hg19; EAF, effective allele frequency; OR, odds ratio; SE, standard error; CSC, central serous chorioretinopathy; AMD, age-related macular degeneration; CFH, complement factor H; LIPC, hepatic lipase; MCUB, mitochondrial calcium uniporter dominant negative subunit beta; PLA2G12A, phospholipase A2 group XIIA; CFI, Complement factor I; APOE, apolipoprotein E; FGD6, FYVE, RhoGEF, and PH domain containing 6; KMT2E, lysine methyltransferase 2E; SRPK2, SRSF protein kinase 2; NOA1, nitric oxide associated 1; REST, RE1 silencing transcription factor; IGFBP7, insulin-like growth factor-binding protein 7; POLR2B, RNA polymerase II subunit B; RORB, RAR-related orphan receptor B; COL10A1, collagen type X alpha 1 chain; ABCA1, ATP-binding cassette subfamily A member 1.*

*^*a*^A1/A2, effective allele and alternative allele.*

**TABLE 2:** Association results for the three SNPs under three different models.

**CHR**	**SNP**	**BP**	**A1/A2^a^**	**Model**	***P*-value**	**OR (95% CI)**
1	rs1410996	196,696,933	A/G	ADD	2.37E−05	1.47 (1.23–1.75)
				DOM	3.22E−02	1.36 (1.03–1.79)
				REC	7.98E−07	2.19 (1.60–3.00)
4	rs4698775	110,590,479	G/T	ADD	2.20E−04	1.45 (1.19–1.76)
				DOM	3.23E−02	1.33 (1.03–1.72)
				REC	2.91E−06	3.08 (1.92–4.93)
15	rs2043085	58,680,954	A/G	ADD	1.91E−04	1.44 (1.18–1.75)
				DOM	4.62E−02	1.37 (1.00–1.87)
				REC	5.90E−05	1.87 (1.37–2.56)

*CHR, chromosome; SNP, single nucleotide polymorphism; BP, base pair; ADD, DOM, REC, additive, dominant, and recessive mode of inheritance, respectively; *P*, the genetic association *p*-values under each mode of inheritance; OR, odds ratio; 95% CI, 95% confidence interval.*

*^*a*^A1/A2, effective allele and alternative allele.*

### Association Analysis of Confirmed CSC Loci With Clinical Phenotypes

**FIGURE 3:**
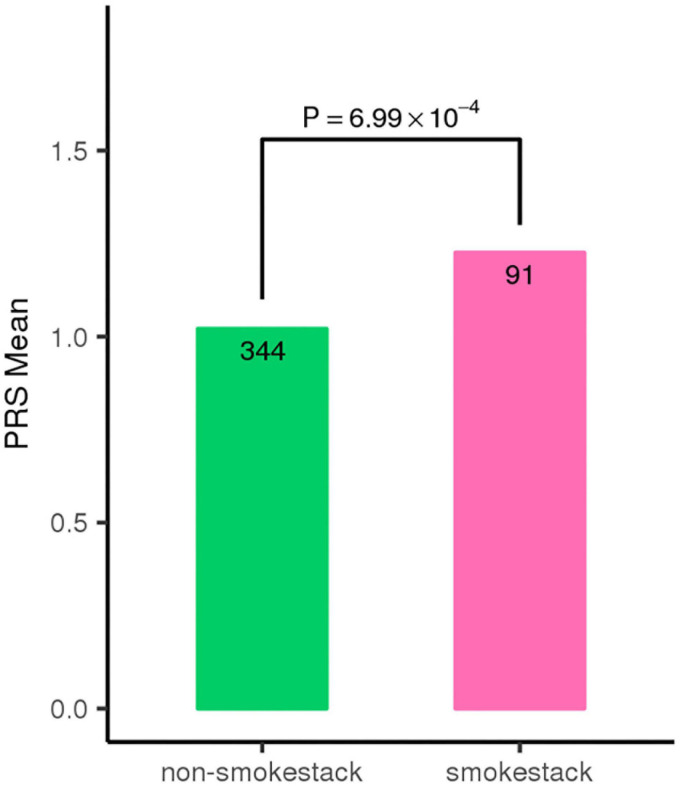
Individuals with classic smoke stack shape of fluid leakage show higher polygenic risk score (PRS) for the top three SNPs.

To evaluate whether these three risk variants have any effect on the clinical manifestations of the disease, we tested the associations of the three SNPs with FFA- and OCT-traits in CSC patients, including the shape, amount and location of fluid leakage, the central subfield thickness and volume, the maximum horizontal and vertical dimensions of SRF. We found that the patients carrying the risk A allele at SNP rs2043085 or the risk G allele at SNP rs4698775 are more likely to exhibit the smokestack shape of fluid leakage than inkblot-like leakage on FFA in acute CSC patients (*OR* = 1.61, *p* = 3.83 × 10^–3^; *OR* = 1.39, *p* = 2.34 × 10^–2^, respectively; Table 3). Consistently, the PRS also showed significant association with a smokestack shape of fluid leakage (*p* = 6.99 × 10^–4^; Figure 3). In addition, the risk allele G of SNP rs1410996 was found to be associated with larger central subfield thickness (β = 0.15, *p* = 2.26 × 10^–2^), increased horizontal dimension (β = 0.14, *p* = 3.78 × 10^–2^), and increased vertical dimension of SRF (β = 0.18, *p* = 7.84 × 10^–3^; Table 4).

**TABLE 3:** Stratified association analysis of three SNPs with shape of fluid leakage on FFA.

**CHR**	**SNP**	**BP**	**A1/A2^a^**	**OR (95% CI)**	***P*-value**	**Trait**
15	rs2043085	58,680,954	G/A	0.62 (0.44–0.87)	3.83E−03	Shape of fluid leakage
4	rs4698775	110,590,479	G/T	1.39 (1.04–1.87)	2.34E−02	Shape of fluid leakage
1	rs1410996	196,696,933	G/A	0.77 (0.57–1.03)	7.78E−02	Shape of fluid leakage

*FFA, fundus fluorescein angiography; CHR, chromosome; SNP, single nucleotide polymorphism; BP, base pair; OR, odds ratio; 95% CI, 95% confidence interval.*

*^*a*^A1/A2, effective allele and alternative allele.*

**TABLE 4:** Association of rs1410996 with OCT manifestation.

**CHR**	**SNP**	**BP**	**A1/A2^a^**	**β^b^**	**SE**	***P*-value**	**Trait**
1	rs1410996	196,696,933	G/A	−0.149	0.065	2.26E−02	Central subfield thickness
1	rs1410996	196,696,933	G/A	−0.140	0.067	3.78E−02	Horizontal dimension of SRF
1	rs1410996	196,696,933	G/A	−0.182	0.068	7.84E−03	Vertical dimension of SRF

*OCT, optical coherence tomography; CHR, chromosome; SNP, single nucleotide polymorphism; BP, base pair in hg19; SE, standard error; SRF, subretinal fluid. ^*a*^Effective allele and alternative allele.*

*^*b*^Effect size of the effective allele on the risk of the related trait.*

### Comparison of the Genetic Effects Between CSC and AMD

**FIGURE 4:**
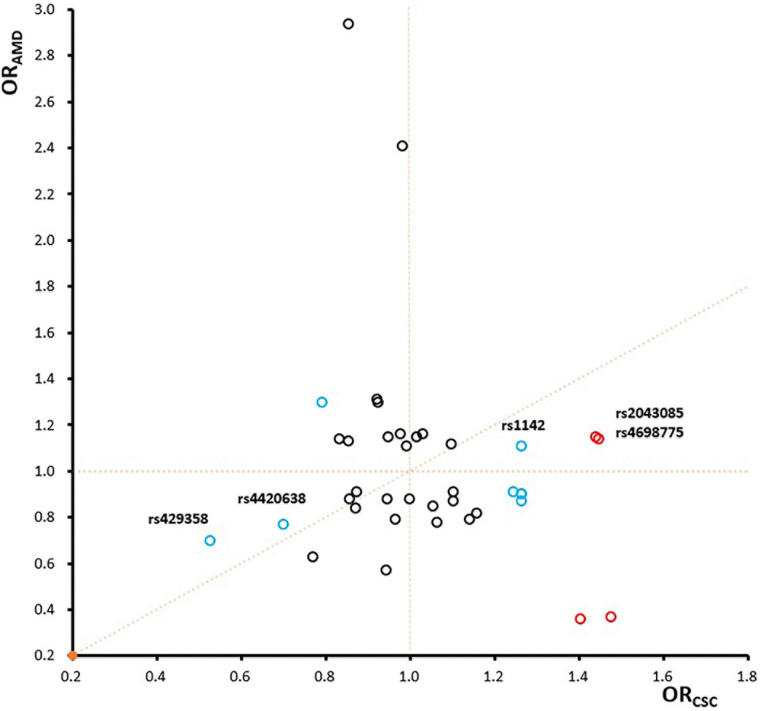
The comparison of odds ratios between CSC and AMD. The four significant SNPs are labeled in red, other eight SNPs with achieved nominal association significance (*p* < 0.05) are labeled in blue, and the remaining ones are in black.

**FIGURE 5:**
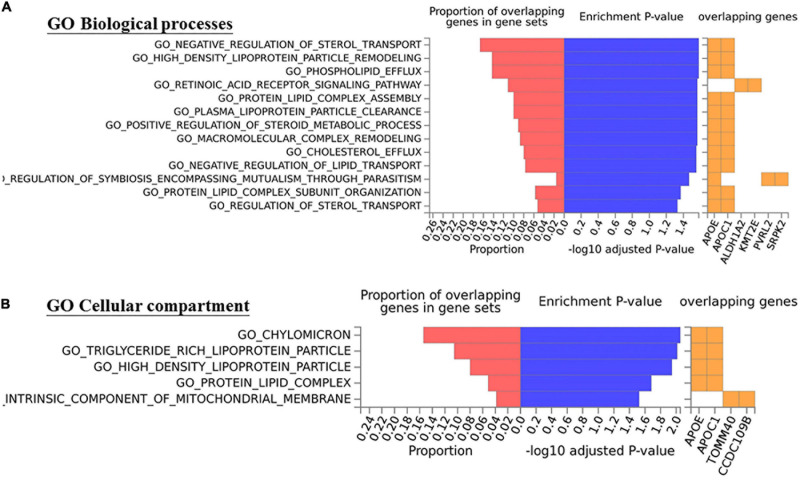
The five SNPs with consistent effectsbetween CSC and AMD show enrichment with Gene Ontology **(A)** Biological processes and **(B)** Cellular compartment terms (mainly related in lipid metabolism, B-H adj *p* < 0.05).

To further investigate genetic pleiotropic effect between CSC and AMD, we compared the estimated genetic effects (ORs) of these 38 SNPs on CSC (estimated from the current study) and the previously published ones on AMD. Of the 12 SNPs that achieved nominal associations in CSC (*p* < 0.05), 7 SNPs had opposite effects between CSC and AMD, while 5 SNPs showed the consensus effects between CSC and AMD (Figure 4). Of the five SNPs with the same direction of effect, four SNPs showed stronger effect in CSC than AMD. Only rs4420638 at *APOE* locus showed consistent effect between CSC (*OR* = 0.70) and AMD (*OR* = 0.77). In addition, we also performed GO term enrichment analysis of these 12 SNPs using FUMA. No significant GO term enrichments were identified for the seven SNPs that showed opposite effects (data not shown). For the 5 SNPs with consistent effects, the analysis revealed 13 significant enriched GO terms (B-H adj *p* < 0.05). These biological pathways and processes are largely driven by the *APOE* and *APOC1* genes and seem to be related to lipid metabolism (Figure 5).

## Discussion

In the current study, the associations of 38 AMD-associated SNPs with the risk of acute CSC were investigated in a large Chinese cohort, consisting of 464 acute CSC patients and 548 healthy controls. Three genetic risk loci (rs1410996 on chromosome 1, rs4698775 on chromosome 4, and rs2043085 on chromosome 15) were identified for CSC with solid evidence that surpass the threshold of statistical significance after Bonferroni correction. Of the three loci, the *CFH* locus on chromosome 1 is a well-established genetic risk locus for CSC, and the other two are novel genetic risk loci for CSC. The top 10% of subjects with highest PRS value showed 6.39 times higher risk than the bottom 10% of subjects with lowest PRS, suggesting that the genetic risk effects of these three risk loci have the potential to identify subjects with high risk of developing acute CSC. These genetic risk variants were also found to impact on the clinic symptoms in CSC patients, further confirming their association with CSC development. To our best knowledge, this is the first genetic association study of acute CSC and the first genetic association study of CSC in Chinese population.

The most significantly association signal was observed at the intronic SNP rs1410996 within the *CFH* gene on chromosome 1. The association of *CFH* genetic variants with chronic CSC has been well-demonstrated by several independent studies (Miki et al., 2014; de Jong et al., 2015; Schellevis et al., 2018). Recently, Mohabati et al. (2019) also confirmed the association between the variants in the *CFH* gene and acute CSC development. The most significant SNP rs1329428 reported in the study of Mohabati et al. (2019) is in strong LD with rs1410996 which was detected in our current study result. Interestingly, we cannot detect any association evidence for rs1061170, although the samples of our current study provided sufficient statistical power for detecting the reported association effect at rs1061170. In addition, SNP rs1061170 are not in LD (*r*^2^ < 0.04) with the two SNPs detected in our current study in our study samples. While it is assuring to see the confirmation of this well-established locus in our Chinese cohort of CSC, our study also revealed the ethnic difference of the *CFH* locus (allelic heterogeneity) between Chinese and Caucasian populations. In addition, for the first time, our study discovered the association of the *CFH* variants with the clinical characteristics of CSC, including shape of fluid leakage, the central subfield thickness, and the horizontal and vertical dimensions of SRF. The *CFH* gene encodes complement factor H, which is a key regulator of complement activation. It has been suggested that it could affect choroidal hemodynamics activities and may lead to RPE damage and dysfunction, which has been proposed to contribute to the pathogenesis of CSC (Guyer et al., 1994; Dorner et al., 2003; de Jong et al., 2015). Together with previous findings, our study has highlighted the potentials of the *CFH* gene variants in terms of acute CSC classification and prediction in future.

We have discovered two novel loci for acute CSC. The first novel locus is SNP rs4698775 that is located in an intron of mitochondrial calcium uniporter dominant negative subunit beta (*MCUB*) gene on chromosome 4. It encodes the protein MCUB, but its function and role in disease development has not been well-studied. Based on the information from GTEx database (Gamazon et al., 2018), rs4698775 showed significant expression quantitative trait loci (eQTL) effect for phospholipase A2 group XIIA (*PLA2G12A*, *p* = 1.20 × 10^–6^ in esophagus mucosa and 4.10 × 10^–5^ in transformed fibroblasts) and *MCUB* (*p* = 1.50 × 10^–6^ in visceral adipose tissue). *PLA2G12A* encodes phospholipase A2 Group XIIA enzyme that is involved in lipid metabolism. It is notable that SNP rs4698775 is proximal to the *CFI* gene. The *CFI* gene encodes complement factor I, with a catalytic serine protease domain that mediates cleavage of C3 protein. C3 is an acute phase protein and highly expressed in the choroid, which may play an important role in the pathogenesis of CSC (Imamura et al., 2009). The second novel locus is SNP rs2043085 on chromosome 15 that is next to *LIPC* gene. Rs2043085 is significantly correlated with LIPC expression level in the GTEx. *LIPC* encodes a hepatic triglyceride lipase that has been shown to be involved in triglyceride hydrolysis and high-density lipoprotein cholesterol (HDL) metabolism and the progression of AMD (Neale et al., 2010). Moreover, our current study also revealed a suggestive association at the *APOE* gene (rs429358, *p* = 8.39 × 10^–3^), which encodes a lipid transport protein. These results suggest that lipid metabolism may play an important role in the development of CSC. The discovery of these two novel loci has advanced the biological understanding of CSC development by not only highlighting the important role of complement system and innate immunity but also revealing the involvement of lipid metabolism in CSC development.

By comparing the genetic risk effects of 38 SNPs for CSC (estimated from the current study) and the previously published ones for AMD, we investigated the genetic pleiotropy between these two diseases. Of the 38 known AMD risk-associated SNPs, 8 SNPs showed nominal associations (*p* < 0.05), and 4 SNPs within 3 loci (*CFH*, *MCUB-PLA2G12A-CFI*, and *LIPC*) showed strong association with acute CSC. The overlapping genetic risk loci between the two diseases strongly suggest that these two diseases share some genetic and pathogenic mechanism. Of the 12 SNPs showing nominal associations in CSC, 7 SNPs showed opposite directions of effects, and 5 SNPs had consistent directions of effects between CSC and AMD. For example, the A allele of rs1410996 and the T allele of rs1329428 within the CFH locus showed protective effects for AMD, but both are risk alleles for acute CSC. In contrast, the C allele of rs1061170 conferred risk for AMD but showed protective effect for CSC. However, the missense mutation Y402H (rs1061170) of *CFH*, which is a strong risk variant for AMD (Zareparsi et al., 2005; Yu et al., 2011) did not show any association effect in our CSC cohort, although our samples provide sufficient statistical power (>80%) for detecting the AMD-associated genetic effect. Interestingly, while we did not see any pathway enrichment for the seven SNPs that showed opposite effects, we did observe the significant enrichment of lipid metabolism (B-H adj *p* < 0.05, mainly driven by *APOE* and *APOC1*) for five SNPs that showed consistent effects between CSC and AMD. Therefore, our study has not only indicated some shared pathophysiologic mechanisms between two diseases but also highlighted the diverse and complex roles of these mechanistic processes in CSC and AMD. In particular, our study suggests that lipid metabolism may play a similar pathogenic role in CSC and AMD, which need to be validated by further functional investigations.

In recent years, several genetic variants have been reported consistently for chronic CSC, mainly in European and Japanese populations (Miki et al., 2014, 2018; Schubert et al., 2014; Breukink et al., 2015; de Jong et al., 2015; van Dijk et al., 2017; Schellevis et al., 2018). Among these studies, the association of the *CFH* gene is consistently replicated for the risk of chronic CSC. In the present study, we validated a significant association of the *CFH* gene for acute CSC, but we were not able to find several other associations that have been found for chronic CSC. For example, SNP rs10490924 within the *ARMS2* gene that has been implicated for the risk of chronic CSC had no significant effect on the risk of acute CSC in our study, which is consistent with previous report (Mohabati et al., 2019). However, because the current study focused on the analysis of AMD-associated risk variants, many previously reported genetic risk loci for chronic CSC were not evaluated. As a limitation, our current study could not investigate potential genetic heterogeneity between chronic and acute CSC. A more thorough investigation, such as an unbiased GWAS would be ideal for novel genetic discovery for acute CSC.

## Conclusion

In conclusion, we have identified three genetic risk loci (i.e., *CFH*, *MCUB-PLA2G12A-CFI*, and *LIPC*) for acute CSC in Chinese population, and *MCUB-PLA2G12A-CFI* and *LIPC* are novel risk loci for CSC. Besides confirming the important role of complement systems and innate immunity, our discovery of novel loci has also suggested the involvement of lipid metabolism in CSC. By demonstrating a good number of shared genetic risk loci between two diseases with both opposite and consistent genetic effects, our study has indicated shared pathophysiological processes with complex functionalities, and in particular, the similar role of lipid metabolism, between the two diseases. Further studies are required to validate the implications of these findings in clinical practice and functional investigations.

## Data Availability Statement

The raw data supporting the conclusions of this article will be made available by the authors, without undue reservation.

## Ethics Statement

The studies involving human participants were reviewed and approved by the institutional review board of the Second Affiliated Hospital of Zhejiang University School of Medicine. The patients/participants provided their written informed consent to participate in this study.

## Author Contributions

YR and LF conceived and designed the experiments. LF, SC, and YR conducted the sample recruitment and quality control. YR, HD, and LF performed the experiments. SC, XY, RD, and JK undertook the data processing and statistical analysis. LF, YR, XY, and JL interpreted the results and wrote the manuscript. All authors contributed to the article and approved the submitted version.

## Conflict of Interest

The authors declare that the research was conducted in the absence of any commercial or financial relationships that could be construed as a potential conflict of interest.

## Publisher’s Note

All claims expressed in this article are solely those of the authors and do not necessarily represent those of their affiliated organizations, or those of the publisher, the editors and the reviewers. Any product that may be evaluated in this article, or claim that may be made by its manufacturer, is not guaranteed or endorsed by the publisher.
