# Supplementary material for: Discovery of Novel Genetic Risk Loci for Acute Central Serous Chorioretinopathy and Genetic Pleiotropic Effect With Age-Related Macular Degeneration
Source: Front Cell Dev Biol. 2021 Aug 20;9:696885. doi: 10.3389/fcell.2021.696885 (PMC8417822; doi:10.3389/fcell.2021.696885)
Supplement: Supplementary file 1 [file Data_Sheet_1.docx]

**Supplement Table1**

The 47 AMD-associated SNPs and their association results with acute CSC in a large Chinese cohort

| SNP | CHR | BP | Gene | A1/A2 | F_A | F_U | SE | P | FDR | OR__CSC_ | OR__AMD_ |
| --- | --- | --- | --- | --- | --- | --- | --- | --- | --- | --- | --- |
| rs1061170 | 1 | 196659237 | CFH | C/T | 0.05 | 0.05 | 0.20 | 9.25E-01 | 9.50E-01 | 0.98 | 2.41^1^ |
| rs1410996 | 1 | 196696933 | CFH | A/G | 0.52 | 0.41 | 0.09 | 2.37E-05 | 9.01E-04 | 1.47 | 0.37^1^ |
| rs1329428 | 1 | 196702810 | CFH | T/C | 0.52 | 0.43 | 0.09 | 3.32E-04 | 3.15E-03 | 1.40 | 0.36^2^ |
| rs6795735 | 3 | 64705365 | ADAMTS9 | C/T | 0.24 | 0.26 | 0.11 | 2.12E-01 | 4.74E-01 | 0.87 | 0.91^3^ |
| rs62247658 | 3 | 64715155 | ADAMTS9 | T/C | 0.23 | 0.25 | 0.11 | 1.55E-01 | 3.95E-01 | 0.86 | 0.88^4^ |
| rs1713985 | 4 | 57786450 | C4orf14, REST, IGFBP7, POLR2B | G/T | 0.23 | 0.27 | 0.11 | 2.86E-02 | 1.21E-01 | 0.79 | 1.30^5^ |
| rs4698775 | 4 | 110590479 | CFI | G/T | 0.29 | 0.21 | 0.10 | 2.20E-04 | 2.79E-03 | 1.45 | 1.14^3^ |
| rs10033900 | 4 | 110659067 | CFI | C/T | 0.38 | 0.36 | 0.09 | 5.77E-01 | 7.56E-01 | 1.05 | 0.85^1^ |
| rs12661281 | 6 | 31842598 | SLC44A4 | A/T | 0.10 | 0.09 | 0.15 | 3.92E-01 | 6.20E-01 | 1.14 | 0.79^6^ |
| rs429608 | 6 | 31930462 | C2-CFB | A/G | 0.06 | 0.07 | 0.20 | 7.55E-01 | 9.25E-01 | 0.94 | 0.57^3^ |
| rs2071277 | 6 | 32171683 | NOTCH4 | C/T | 0.36 | 0.38 | 0.10 | 4.08E-01 | 6.20E-01 | 0.92 | 1.30^7^ |
| rs943080 | 6 | 43826627 | VEGFA | C/T | 0.31 | 0.32 | 0.10 | 9.76E-01 | 9.76E-01 | 1.00 | 0.88^4^ |
| rs2295334 | 6 | 43970827 | C6orf223 | A/G | 0.26 | 0.24 | 0.10 | 5.59E-01 | 7.56E-01 | 1.06 | 0.78^6^ |
| rs3812111 | 6 | 116443735 | COL10A1 | A/T | 0.25 | 0.19 | 0.11 | 4.12E-02 | 1.42E-01 | 1.24 | 0.91^3^ |
| rs1142 | 7 | 104756326 | KMT2E, SRPK2 | T/C | 0.33 | 0.29 | 0.10 | 2.30E-02 | 1.21E-01 | 1.26 | 1.11^4^ |
| rs13278062 | 8 | 23082971 | TNFRSF10A | T/G | 0.31 | 0.30 | 0.10 | 8.87E-01 | 9.50E-01 | 1.01 | 1.15^3^ |
| rs10781182 | 9 | 76617720 | MIR6130, RORB | G/T | 0.29 | 0.24 | 0.11 | 4.11E-02 | 1.42E-01 | 1.26 | 0.90^4^ |
| rs334353 | 9 | 101908365 | TGFBR1 | G/T | 0.40 | 0.42 | 0.09 | 5.36E-01 | 7.54E-01 | 0.94 | 0.88^3^ |
| rs2740488 | 9 | 107661742 | ABCA1 | C/A | 0.26 | 0.22 | 0.12 | 4.98E-02 | 1.58E-01 | 1.26 | 0.90^4^ |
| rs10490924 | 10 | 124214448 | ARMS2/HTRA1 | T/G | 0.37 | 0.42 | 0.10 | 9.73E-02 | 2.84E-01 | 0.85 | 2.94^1^ |
| rs10507047 | 12 | 95604290 | FGD6 | C/T | 0.34 | 0.29 | 0.10 | 1.85E-02 | 1.17E-01 | 1.26 | 0.87^6^ |
| rs9564692 | 13 | 31821240 | B3GALTL | C/T | 0.27 | 0.26 | 0.10 | 3.74E-01 | 6.20E-01 | 1.10 | 1.12^4^ |
| rs8017304 | 14 | 68785077 | RAD51B | A/G | 0.41 | 0.41 | 0.09 | 9.13E-01 | 9.50E-01 | 0.99 | 1.11^3^ |
| rs10468017 | 15 | 58678512 | LIPC | T/C | 0.16 | 0.18 | 0.13 | 2.63E-01 | 5.26E-01 | 0.87 | 0.84^1^ |
| rs2043085 | 15 | 58680954 | LIPC | A/G | 0.55 | 0.46 | 0.10 | 1.91E-04 | 2.79E-03 | 1.44 | 1.15^4^ |
| rs493258 | 15 | 58687880 | LIPC | G/A | 0.22 | 0.22 | 0.11 | 8.17E-01 | 9.25E-01 | 0.97 | 1.16^8^ |
| rs920915 | 15 | 58688467 | LIPC | C/G | 0.19 | 0.22 | 0.11 | 1.56E-01 | 3.95E-01 | 0.85 | 1.13^3^ |
| rs2070895 | 15 | 58723939 | LIPC | A/G | 0.42 | 0.40 | 0.09 | 3.07E-01 | 5.79E-01 | 1.10 | 0.87^4^ |
| rs3764261 | 16 | 56993324 | CETP | A/C | 0.16 | 0.16 | 0.13 | 6.60E-01 | 8.36E-01 | 0.95 | 1.15^1^ |
| rs17231506 | 16 | 56994528 | CETP | T/C | 0.16 | 0.16 | 0.13 | 8.28E-01 | 9.25E-01 | 1.03 | 1.16^4^ |
| rs1864163 | 16 | 56997233 | CETP | A/G | 0.19 | 0.16 | 0.12 | 2.39E-01 | 5.05E-01 | 1.16 | 0.82^3^ |
| rs72802342 | 16 | 75234872 | CTRB2, CTRB1, BCAR1 | A/C | 0.10 | 0.11 | 0.15 | 8.05E-01 | 9.25E-01 | 0.96 | 0.79^4^ |
| rs11080055 | 17 | 26649724 | TMEM97, VTN | A/C | 0.30 | 0.27 | 0.10 | 3.20E-01 | 5.79E-01 | 1.10 | 0.91^4^ |
| rs429358 | 19 | 45411941 | APOE | C/T | 0.03 | 0.06 | 0.24 | 8.39E-03 | 6.38E-02 | 0.53 | 0.70^4^ |
| rs4420638 | 19 | 45422946 | APOE | G/A | 0.08 | 0.11 | 0.16 | 2.56E-02 | 1.21E-01 | 0.70 | 0.77^3^ |
| rs5749482 | 22 | 32663679 | TIMP3 | G/C | 0.27 | 0.28 | 0.11 | 4.31E-01 | 6.30E-01 | 0.92 | 1.31^3^ |
| rs9621532 | 22 | 32688525 | TIMP3 | C/A | 0.02 | 0.03 | 0.31 | 3.91E-01 | 6.20E-01 | 0.77 | 0.63^1^ |
| rs8135665 | 22 | 38476276 | SLC16A8 | T/C | 0.11 | 0.13 | 0.14 | 2.00E-01 | 4.74E-01 | 0.83 | 1.14^4^ |
| rs11884770 | 2 | 227222204 | COL4A3 | C/T | - | - | - | - |  | - | 0.90^4^ |
| rs13081855 | 3 | 99762695 | COL8A1-FILIP1L | T/G | - | - | - | - |  | - | 1.23^3^ |
| rs3130783 | 6 | 30806580 | IER3-DDR1 | A/G | - | - | - | - |  | - | 1.16^3^ |
| rs4711751 | 6 | 43860845 | VEGFA | C/T | - | - | - | - |  | - | 1.15^1^ |
| rs3138141 | 12 | 55721994 | RDH5, CD63, MMP19 | A/C | - | - | - | - |  | - | 1.16^4^ |
| rs9542236 | 13 | 31245188 | B3GALTL | C/T | - | - | - | - |  | - | 1.10^3^ |
| rs6565597 | 17 | 81559795 | NPLOC4, TSPAN10 | T/C | - | - | - | - |  | - | 1.13^4^ |
| rs67538026 | 19 | 1031439 | CNN2, GPX4 | T/C | - | - | - | - |  | - | 0.90^4^ |
| rs2230199 | 19 | 6718376 | C3 | C/G |  |  |  |  |  |  | 1.43^4^ |

Abbreviations: SNP, single nucleotide polymorphism; CHR, chromosome; BP, base pair in hg19; F_A, allele frequency of cases; F_U, allele frequency of controls; FDR,false discovery rate; SE, standard error; OR, odds ratio; CSC, central serous chorioretinopathy; AMD, age-related macular degeneration;

Reference

1. Yu, Y. *et al.* Common variants near FRK/COL10A1 and VEGFA are associated with advanced age-related macular degeneration. *Hum Mol Genet* **20**, 3699-709 (2011).

2. Kopplin, L.J. *et al.* Genome-wide association identifies SKIV2L and MYRIP as protective factors for age-related macular degeneration. *Genes Immun* **11**, 609-21 (2010).

3. Fritsche, L.G. *et al.* Seven new loci associated with age-related macular degeneration. *Nat Genet* **45**, 433-9, 439e1-2 (2013).

4. Fritsche, L.G. *et al.* A large genome-wide association study of age-related macular degeneration highlights contributions of rare and common variants. *Nat Genet* **48**, 134-43 (2016).

5. Arakawa, S. *et al.* Genome-wide association study identifies two susceptibility loci for exudative age-related macular degeneration in the Japanese population. *Nat Genet* **43**, 1001-4 (2011).

6. Cheng, C.Y. *et al.* New loci and coding variants confer risk for age-related macular degeneration in East Asians. *Nat Commun* **6**, 6063 (2015).

7. Cipriani, V. *et al.* Genome-wide association study of age-related macular degeneration identifies associated variants in the TNXB-FKBPL-NOTCH4 region of chromosome 6p21.3. *Hum Mol Genet* **21**, 4138-50 (2012).

8. Neale, B.M. *et al.* Genome-wide association study of advanced age-related macular degeneration identifies a role of the hepatic lipase gene (LIPC). *Proc Natl Acad Sci U S A* **107**, 7395-400 (2010).
